# NOL7 facilitates melanoma progression and metastasis

**DOI:** 10.1038/s41392-021-00676-3

**Published:** 2021-10-13

**Authors:** Yumei Li, Chunlian Zhong, Jie Wang, Fan Chen, Weiyu Shen, Bifei Li, Ning Zheng, Yusheng Lu, Vladimir L. Katanaev, Lee Jia

**Affiliations:** 1 Institution of Oceanography, Minjiang University, Fuzhou, Fujian China; 2 Cancer Metastasis Alert and Prevention Center, Fujian Provincial Key Laboratory of Cancer Metastasis Chemoprevention and Chemotherapy, College of Chemistry, Fuzhou University, Fuzhou, Fujian China; 3 School of Basic Medicine, Gannan Medical University, Ganzhou, Jiangxi China; 4 School of Pharmacy, Anhui Medical University, Hefei, Anhui China; 5 Department of Pharmacology, Fujian Key Laboratory of Natural Medicine Pharmacology, School of Pharmacy, Fujian Medical University, Fuzhou, Fujian China; 6 Natural Products Drug Discovery Laboratory, School of Biomedicine, Far Eastern Federal University, Vladivostok, Russia; 7 Department of Cell Physiology and Metabolism, Translational Research Center in Oncohaematology, Faculty of Medicine, University of Geneva, Geneva, Switzerland

**Keywords:** Cell biology, Skin cancer, Molecular biology

**Dear Editor**,

Metastasis is the cause of most fatalities in cancer patients and remains the phenomenon poorly understood mechanistically. Deciphering of the regulatory networks underlying the cancer cell metastasis is urgently needed. Nucleolar protein 7 (NOL7) has been reported to function as a tumor suppressor in cervical cancer.^1^ Our current study reveals a novel tumor-promoting capacity of NOL7 in melanoma. We first detected that NOL7 expression is upregulated in metastatic melanoma as compared with its expression at the primary site through isobaric tag for relative and absolute quantitation proteomic screening, further confirming this finding with the analysis of NOL7 protein and messenger RNA (mRNA) levels (Supplementary Fig. S1). Importantly, NOL7 expression increased with the disease progression from benign nevus to primary melanoma and further to metastatic melanoma (Fig. 1a and Supplementary Fig. S1d). Previous studies have shown that melanoma is commonly associated with the amplification of the chromosome region 6p, particularly 6p21–23, where the *NOL7* gene resides, and that this region frequently undergoes heterozygous loss in cervical cancer.^2^ It might therefore be predicted that NOL7 exhibits a different expression pattern and plays different roles in melanoma and cervical cancer.

**Fig. 1:**
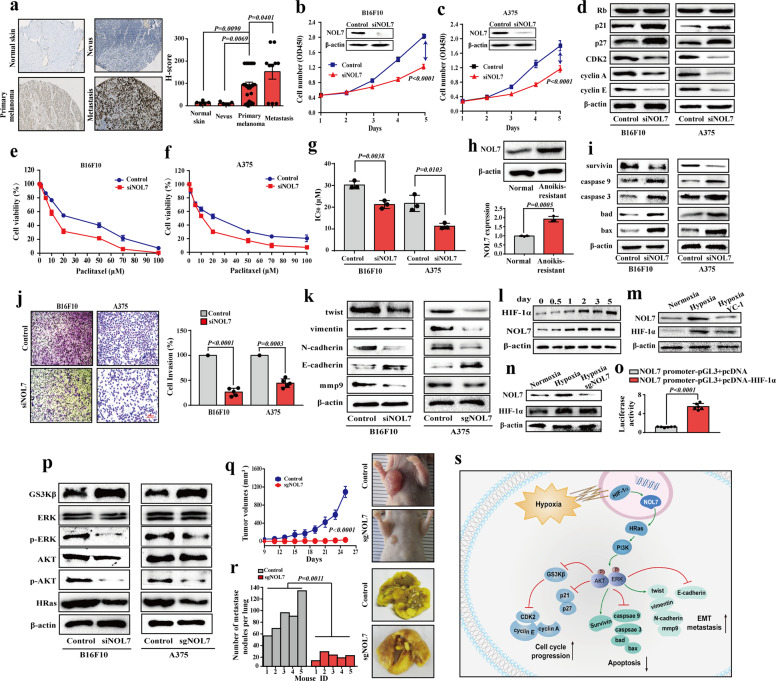
NOL7 facilitates melanoma progression and metastasis. **a** NOL7 expression increases in the disease progression from normal skin and nevus to primary melanoma and further to metastatic melanoma as detected by IHC (left panels). The *H*-score for the quantification of NOL7 expression is shown on the right panel. **b**, **c** Lower cell proliferation upon NOL7 knockdown in B16F10 (**b**) and A375 (**c**) cells. Insets illustrate strongly decreased NOL7 levels upon siRNA targeting of NOL7. **d** Effect of NOL7 knockdown on cell cycle-associated proteins in B16F10 and A375 cells. NOL7 knockdown decreased the expression level of cyclin A, cyclin E, CDK2, and enhanced the expression level of p21 and p27. See Supplementary Fig. S2g, h for quantification. **e**–**g** NOL7 knockdown increased sensitivity of B16F10 (**e**) and A375 (**f**) cells to paclitaxel. Target cells were incubated with paclitaxel at various concentrations for 24 h and cell viability was tested by the MTT assay. Quantification of IC_50_ values is shown in (**g**). **h** Western blotting shows the upregulation of NOL7 expression in anoikis-resistant A375 cells as compared to normal A375 cells. Quantitative analysis of NOL7 expression is shown below. **i** Effect of NOL7 knockdown on apoptosis-related proteins in B16F10 and A375 cells. NOL7 knockdown decreased the expression level of survivin and increased the expression level of caspase 3, caspase 9, bad, and bax. See Supplementary Fig. S5b, c for quantification. **j** Phase micrographs showing the invading B16F10 and A375 cells (control and after NOL7 knockdown) at different time intervals. Quantitative analysis of the effect of NOL7 knockdown on cell invasion of B16F10 and A375 cells is shown on the right. **k** Effect of NOL7 knockdown on EMT-associated proteins in B16F10 and A375 cells. NOL7 depletion increased the expression level of epithelial marker E-cadherin and decreased the expression level of mesenchymal markers, N-cadherin, mmp9, vimentin, and twist in B16F10 and A375 cells. See Supplementary Fig. S6c, d for quantification. **l**–**n** Western blotting shows the concurrent NOL7 and HIF-1α expression under hypoxia (**l**) plus YC-1 (HIF-1α inhibitor) (**m**) or upon NOL7 depletion (**n**). See Supplementary Fig. 9b, c for quantification. **o** HIF-1α induces NOL7 expression through promoting the NOL7 transcription, as measured in a dual-luciferase reporter assay following transient transfection with the NOL7 promoter-pGL3 plasmid and pcDNA-HIF-1α (or the empty pcDNA). The pRL-TK vector was co-transfected to normalize transfection efficiencies. **p** NOL7 depletion suppressed the PI3K/AKT/ERK signaling pathway through inhibiting the AKT and ERK phosphorylation and activating GSK3β. See Supplementary Fig. S10a, b for quantification. **q** Dramatically reduced tumor growth in nude mice injected with NOL7-knockout cells, compared with that in the control group. Representative images of tumors are shown on the right. **r** NOL7 knockout strongly decreased the number of lung metastatic nodules. Representative photographs of pulmonary tumor metastases are shown on the right. **s** Schematic illustration displays the mechanism of NOL7 promoting melanoma tumorigenesis and metastasis. Throughout the figure, data are presented as mean ± s.d. (*n* ≥ 3); *p* values are calculated using the *t* test

To investigate the effects of NOL7 in diverse melanoma activities, we suppressed NOL7 expression by specific small interfering RNAs and the CRISPR/Cas9 gene-editing system in melanoma cell lines B16F10 and A375. With this approach, we observed that NOL7 knockdown/knockout significantly reduced the proliferative abilities, including the cell proliferation rate (Fig. 1b, c and Supplementary Fig. S2b, d), cell colony formation (Supplementary Fig. S2a, c), and cell cycle progression (Supplementary Fig. S2e, f) in both cell lines. These effects of NOL7 knockdown correlated with the decreased levels of the cell cycle regulators CDK2, cyclin A, and cyclin E, and with the reciprocally increased levels of the cell cycle progression inhibitors p21 and p27 (Fig. 1d and Supplementary Fig. S2g, h).

We then moved to study the potential role of NOL7 in melanoma cell apoptosis and stress resistance. Apoptosis assays based on Annexin V-FITC/PI and JC-1 staining indicated a higher apoptotic index in the NOL7-knockdown/knockout cells (Supplementary Figs. S3 and 4). Further, the MTT assay showed a higher sensitivity of the NOL7-knockdown cells to paclitaxel, as compared with the control melanoma cells (Fig. 1e–g). We also domesticated anoikis-resistant A375 cells in a continuous cycle between adherent and suspended culture conditions, finding a significant upregulation of NOL7 expression upon acquisition of resistance to the anoikis (Fig. 1h). Accordingly, NOL7 depletion impaired anoikis resistance of the melanoma cells (Supplementary Fig. S5a). Consistently, NOL7 knockdown in B16F10 and A375 cells markedly reduced the expression of survivin, while stimulating the expression of apoptotic caspase-3, caspase-9, bad, and bax (Fig. 1i and Supplementary Fig. S5b, c). Cumulatively, these results indicate the importance of melanoma NOL7 in the regulation of apoptosis and in cell adaptation and protection against stress conditions, such as chemotherapy or anoikis.

Next, we moved to study the function of NOL7 in metastasis. Upon NOL7 knockdown, the motility, adhesiveness, migration, and invasiveness of B16F10 and A375 cells were significantly decreased (Fig. 1j and Supplementary Fig. S6a, b). Re-activation of NOL7 expression in A375-sgNOL7 cells led to the restoration of these metastatic behaviors. Upon NOL7 overexpression, the invasiveness (but not migration or adhesiveness) of A375 cells was significantly increased (Supplementary Fig. S7). Further, NOL7 depletion increased the expression of the epithelial marker E-cadherin and decreased the levels of mesenchymal markers N-cadherin, mmp9, vimentin, and twist in B16F10 and A375 cells (Fig. 1k and Supplementary Fig. S6c, d). Cumulatively, these findings demonstrate by multiple means that NOL7 plays crucial roles in aggressive behaviors of melanoma in vitro.

Further to examine the role of NOL7 in melanoma growth and metastasis in vivo, we used the A375-sgNOL7 cells and control cells to establish a subcutaneous tumor mouse model and an experimental metastasis model. We found that NOL7 knockout markedly reduced the subcutaneous tumor growth and pulmonary metastatic nodules in nude mice (Fig. 1q, r); immunohistochemical staining confirmed the essentially undetectable NOL7 expression in the tumors formed by the A375-sgNOL7 cells (Supplementary Fig. S8a, b). Further, NOL7 was observed to be associated with the overall survival of mice in the experimental metastasis model (Supplementary Fig. S8c).

Having determined the importance of NOL7 for the multiple aspects of melanoma progression, from tumor cell proliferation to metastasis, we then set to unravel the potential molecular mechanisms behind it. Western blotting was used to test the expression of hypoxia-inducible factor-1α (HIF-1α) and NOL7 in A375 cells exposed to hypoxic conditions (1% O_2_) for different durations. Remarkably, we found that like HIF-1α, the NOL7 expression accumulated in response to hypoxia (Fig. 1l and Supplementary Fig. S9a). Interestingly, NOL7 expression was induced by hypoxia with a delay as compared to HIF-1α, peaking at 2 days; stable NOL7 expression was maintained up to 5 days (Supplementary Fig. S9a). The delay in NOL7 expression under hypoxia could indicate that NOL7 was induced by HIF-1α. To test this possibility, we employed YC-1, a chemical compound that accelerates HIF-1α degradation and blocks HIF-1 expression,^3^ during 2 days of culturing under hypoxic (1% O_2_) conditions. As a result, the protein levels of NOL7 and HIF-1α returned to their initial levels (Fig. 1m and Supplementary Fig. S9b). Upon knockout of NOL7 in A375 cells, hypoxia still led to increased HIF-1α expression (Fig. 1n and Supplementary Fig. S9c), further agreeing with the hypoxia–HIF-1α–NOL7 axis. In order to provide a direct evidence in favor of this axis, we further determined the transcriptional activity of HIF-1α on the NOL7 promoter sequence using a dual-luciferase reporter assay. Indeed, HIF-1α could induce NOL7 expression through promoting the NOL7 transcription (Fig. 1o).

In addition, culturing A375 cells under hypoxic conditions promoted conversion of the nonpolar epithelial phenotype to the polar mesenchymal phenotype, and altered the epithelial–mesenchymal transition (EMT)-associated protein expression (E-cadherin, N-cadherin, vimentin, and fibronectin). However, NOL7 depletion reversed the hypoxia-induced EMT (Supplementary Fig. S9d, e). A375 cells’ invasion, migration, and chemoresistance to paclitaxel were strikingly enhanced after the hypoxic treatment, while NOL7 knockout impaired the elevated cell invasion and migration characteristics and the chemoresistance (Supplementary Fig. S9f–i). Thus, NOL7 is induced by HIF-1α under hypoxia and is required for the HIF-1α-induced EMT, invasiveness, and chemoresistance. Further exploring the molecular mechanism(s) of the NOL7-induced melanoma malignancy, we found that NOL7 knockdown/knockout in B16F10 and A375 cells led to decreased levels of p-AKT and p-ERK, while GSK3β, a negative regulator of the PI3K/AKT/ERK signaling and the Wnt/β-catenin signaling,^4,5^ was upregulated (Fig. 1p and Supplementary Fig. S10a, b). Given the known nucleolar activities of NOL7, one could envisage a potential influence of NOL7 on the dynamics of ribosome production, which may indirectly control the PI3K/AKT/ERK signaling. In addition, we wondered whether the established RNA-binding capacity of NOL7 (see “Methods”) might explain its effect on the PI3K/AKT/ERK pathway and turned to RNAct, a comprehensive whole-genome database of predicted protein–RNA interactions. Out of several RNA targets predicted to interact with NOL7 (rnact.crg.eu/protein?query=Q9UMY1), the mRNA for HRas being close to the top of the prediction score attracted our attention. We hypothesized that NOL7 could influence the HRas mRNA translation efficiency, thus regulating the HRas protein levels and the PI3K/AKT/ERK signaling pathway. Indeed, we found that NOL7 depletion markedly decreased HRas protein levels in B16F10 and A375 melanoma cells (Fig. 1p and Supplementary Fig. S10a,b), while the HRas transcript levels were not affected (Supplementary Fig. S10c). While the exact mode of action of NOL7 on HRas mRNA remains to be investigated, our findings suggest that NOL7 controls HRas production post-transcriptionally, thus affecting the intensity of the PI3K/AKT/ERK signaling pathway.

Taken together, our research reveals that NOL7 exerts pro-oncogenic effects on melanoma progression and metastasis. From the functional perspective, NOL7 is beneficial for cell survival and contributes to tumor growth and metastasis of melanoma by promoting cell proliferation, cell cycle progression, and aggressiveness, as well as the acquisition of chemo- and anoikis resistance. From a mechanistic perspective, NOL7 emerges as a novel player within the HIF-1α/NOL7/HRas/PI3K/AKT/ERK axis to ultimately activate regulators of the cell cycle, apoptosis, and EMT to exert its oncogenic function (Fig. 1r). Therefore, we identify NOL7 as a multifunctional regulator of malignant activities, proposing it as a novel biomarker of melanoma progression and potential drug target for future therapeutics.

## Supplementary information

Supplementary information

## Data Availability

The data used for the current study are available from the corresponding author upon reasonable request.
