# Supplementary material for: NOL7 facilitates melanoma progression and metastasis
Source: Signal Transduct Target Ther. 2021 Oct 13;6:352. doi: 10.1038/s41392-021-00676-3 (PMC8511122; doi:10.1038/s41392-021-00676-3)
Supplement: Supplementary file 1 — Supplementary information [file 41392_2021_676_MOESM1_ESM.pdf]

Supplementary Materials for

NOL7 facilitates melanoma progression and metastasis

Yumei Li[1,2,5#], Chunlian Zhong[1#], Jie Wang[2,7], Fan Chen[2], Weiyu Shen[2], Bifei Li[2], Ning Zheng[6], Yusheng Lu[1], Vladimir L. Katanaev[1,3,4*], Lee Jia[1,2*]

[1]Institution of Oceanography, Minjiang University, Fuzhou, Fujian 350108, China.
[2]Cancer Metastasis Alert and Prevention Center, Fujian Provincial Key Laboratory of Cancer Metastasis Chemoprevention and Chemotherapy, College of Chemistry, Fuzhou University, Fuzhou, Fujian 350108, China.
[3]Natural Products Drug Discovery Laboratory, School of Biomedicine, Far Eastern Federal University, Vladivostok, Russia.
[4]Translational Research Center in Oncohaematology, Department of Cell Physiology and Metabolism, Faculty of Medicine, University of Geneva, Switzerland.
[5]School of Basic Medicine, Gannan Medical University, Ganzhou, Jiangxi 341000, China
[6]Department of Pharmacology, Fujian Key Laboratory of Natural Medicine Pharmacology, School of Pharmacy, Fujian Medical University, Fuzhou, Fujian 350122, China
[7]School of Pharmacy, Anhui Medical University, Hefei, Anhui, 230000, China

# These authors contributed equally to this work.

* **Corresponding to Lee Jia** (cmapcjia1234@163.com) and **Vladimir L. Katanaev** (Vladimir.Katanaev@unige.ch)

This PDF file includes:

Materials and Methods

Figures. S1 to S10

Table. S1

Other Supplementary Materials for this manuscript include the following:

None.

Materials and Methods

In Silico Analysis

Microarray analysis dataset for NOL7 expression in human melanoma, metastatic melanoma and benign nevus tissues was obtained from the gene expression profiles, GSE3189 and GSE4587 which were accessed freely in Gene Expression Omnibus (GEO) repository (ncbi.nlm.nih.gov/geo/).

To identify mRNAs that can interact with NOL7, an established RNA-binding protein[1,2], we used RNAct, a comprehensive whole-genome database of predicted protein-RNA interactions[3]. Out of several RNA targets predicted to interact with NOL7 (rnact.crg.eu/protein?query=Q9UMY1), the mRNA for HRas was close to the top of the predicted targets (prediction score = 36.11).

Cell, cell culture and establishment of pulmonary metastasis cell lines

The mouse melanoma cell line B16F10 and the human melanoma cell line A375 were procured from Cell Resource Center of the Shanghai Institute for Biological Sciences (Chinese Academy of Sciences, Shanghai, China). Established post-metastasized melanoma cells, B16F10M and A375M were derived from the pulmonary metastases formed in mice by tail vein injection of pro-metastasized parental B16F10 and A375 cells, respectively, via a primary cell line establishment method[4,5]. This experimental metastasis refers to the injection of tumor cells directly to the systemic circulation and circumvents the invasion and extravasion.

The cells mentioned above were cultured in RPMI 1640 medium (Hyclone, Logan, UT, USA). Human pulmonary microvascularendothelial cells (HPMECs) were purchased from Cell Resource Center of the Shanghai Institute for Biological Sciences and cultured in ECM medium (Hyclone). All culture media was supplemented with 10 % (v/v) fetal bovine serum (FBS, Gemini Bioproducts, West Sacramento, CA) and 100 μg/mL penicillin/streptomycin (Sigma-Aldrich, St Louis, USA). Cells were maintained in a humidified atmosphere with 5 % CO_2 at 37°C. Low oxygen concentration (1 % O2) was controlled by 245 whitley H35 HEPA hypoxystation (UK) or treated with YC-1 (MedChem Express, Princeton, NJ, USA).

The cell lines were authenticated by Genetic Testing Biotechnology Corporation (Suzhou, China) using short tandem repeat (STR) markers. Cells were collected with 0.25 % trypsin (Hyclone) and counted by the ADAM MC Auto Cell Counter (NanoEnTek, Korea) before used.

Antibodies and reagents

The following antibodies were used: anti-NOL7 (#DF9720), anti-p-Smad2/3 (#AF3367) from Affinity Biosciences (Cincinnati, OH, USA); anti-PCNA (#PC10), anti-E-cadherin (#3195), anti-N-cadherin (#13116), anti-vimentin (#5741), anti-β-catenin (#8480), anti-p21 (#2947), anti-p27 (#3686), anti-CDK2 (#2546), and anti-mmp9 (#13667), anti-Smad2/3 (#D7G7) from Cell Signaling Technologies (Beverly, MA, USA); anti-AKT 1/2/3 (#sc8312), anti-p-AKT 1/2/3 (Ser 473, #sc33437), anti-ERK 1/2 (#sc135900), anti-p-ERK 1/2 (Thr 202/Tyr 204, #sc16982) and anti-twist (#sc81417) from Santa Cruz Biotechnology Inc. (Santa Cruz, CA); anti-JNK (#WL01295), anti-p-JNK (#WL01295), anti-HIF-1α (#WL01607), anti-Gsk3β (#WL0146), anti-caspase 3 (#WL02117), anti-caspase 9 (#WL01551), anti-bad (#WL02304), anti-bax (#WL01637), anti-survivin (#WL03492), anti-cyclin A (#WL01841) and anti-cyclin E (#WL01072) from Wanlei Biotechnology (Shenyang, China); anti-fibronectin (#40932) from SAB Technology (Danvers, USA); anti-TSP-1 (#A2125), anti-Rb (#A3618), anti-HRas (#A7901) from ABclonal Technology (Wuhan, China); β-actin (#AC001-R), secondary antibody goat anti-mouse-IgG horseradish peroxidase and goat anti-rabbit-IgG horseradish peroxidase from Dingguochangsheng Biotechnology (Beijing, China).

NOL7 siRNA (sense, 5’-3’, GCUGUAUUAGAGCAGCUAATT) for B16F10 cells, NOL7 siRNA (sense, 5’-3’, GGAAAUGACUCCAAGAAAGATT) for A375 cells, corresponding negative control siRNA (UUCUCCGAACGUGUCACGUTT), NOL7 guide RNA sequences (sgRNA, sense, 5’-3’, CACCGACCGCGAGCGTCTCGCGCCC) and NOL7 expression plasmids containing NOL7 cDNA (NM_001317724.1) for A375 cells were synthesized by Sangon Biotech (Shanghai, China).

Gene constructs and generation of stable transfectants

Specific siRNAs were used for the transient knockdown of NOL7 in B16F10 and A375 cells. CRISPR/Cas9 system was operated to generate the stable NOL7 knockout A375 cells, named (A375-sgNOL7 cells) based on a previous protocol[6]. In short, sgRNA for A375 cells was designed by a CRISPR design platform (Zhang Feng Lab, http://crispr.mit.edu/) and then cloned into the PX458 vector. The empty vector was used as a control, named (A375-control cells). These oligo duplexes were transfected into cells via Lipofectamine 3000 Kit (Invitrogen, MA, USA) through standard procedure. Furthermore, stable NOL7-knockout cells were sorted through GFP-activated cell sorting method by flow cytometer (FACS Aria III, BD, San Diego, USA). The pCDH-CMV-EF1-copGFP vector containing the human NOL7 cDNA sequence (NM_001317724.1) was produced by Sangon Biotech. Similarly, constructed overexpression vectors were transfected into A375 cells and stable NOL7-overexpression cells (named A375-overNOL7 cells) were sorted out by flow cytometry. Forced NOL7 knockout and overexpression was validated through western blotting.

Cell proliferation assay

For cell growth kinetic assay, NOL7-knockdown melanoma cells or control cells were plated in 96-well plates (5×10^3 cells/well). Cell proliferation was monitored every day for 5 days using cell counting kit-8 assay (CCK8, Promega, Sunnyvale, CA) according to the manufacturer's protocol.

For cell spherical growth assay, A375-sgNOL7 cells or control cells were put into 96-well microplates with ultralow attachment surfaces (Corning). Visually 3D cell spheroids were formed after 24 h, and their growth was observed and photographed at regular time intervals by phase contrast microscopy to monitor the tumor spheres diameter.

Colony formation assay

A total of 1000 target cells per well were plated in 6-well plates. After culture in complete medium for 14 days, cell colonies were washed three times with PBS and fixed in 4 % paraformaldehyde for 20 min and then, stained with a 0.2 % crystal violet (Sigma-Aldrich) solution for 40 min at room temperature. An overall image of the cell colonies was mapped by Canon scanner (Shanghai, China). Colonies with 50 or more number of cells were counted under a light microscope (Zeiss, Oberkochen, Germany).

Cell cycle assay

Suspended cells were washed twice with precooled PBS and fixed in 70 % ethanol for 24 h at 4°C. The cells were then stained with a fluorescent solution (1 % (v/v) Triton X-100, 0.01 % RNase, 0.05 % PI (Sigma-Aldrich)) for 30 min at 37°C in the dark. Ten thousand events were acquired by flow cytometry to measure the cell cycle distribution. Data were processed by Modfit software 3.2.

Cell apoptosis assay

Using an Annexin V-FITC/PI kit (KeyGen Biotech, China), cell apoptosis was detected by flow cytometry. Target cells at a density of 3×10^5 cells per well were plated in 6-well plates in triplicate. The cells were preprocessed under the following conditions: one group of cells was cultured in complete medium; one group of cells was treated with 10 μM paclitaxel (Meilunbio, Dalian, China) and another group of cells was kept in a suspended state or in FBS-free medium. All of these cells were collected separately after 24 h, and stained with Annexin V-FITC/PI for 15 min under the lucifuge condition. At least 10^4 cells from each specimen were examined by flow cytometry and data were analyzed by FlowJo software 10.0. In addition, using an JC-1 kit (Beyotime, China), cell apoptosis was measured by mitochondrial membrane potential (△Ψm) through an inverted fluorescence microscope (Zeiss). Along the same cell culture conditions as above, cells were stained with 5 μM of JC-1 for 20 min at 37°C. After washing, photographic images were acquired. Red fluorescence

indicates JC-1 monomers (high △Ψm) aggregated in the mitochondria, and green indicates JC-1 aggregates (low △Ψm) scattered in the cytoplasm.

Domestication of anoikis-resistant cells

Anoikis-resistant cells were generated through continuous cycle of culturing on normal (adherent) and soft agar (Sigma-Aldrich) -coated cell culture well (suspended). Briefly, target cells were plated in soft agar-coated wells and maintained under suspended conditions for the indicated time intervals. After 24 h incubation, cells were washed and transferred to normal cell culture wells. We measured the anoikis-resistance of cells by analyzing the cells apoptosis.

In vitro cytotoxicity assay

A MTT (3-(4,5-dimethylthiazol-2-yl)-2, 5-diphenyltetrazolium bromide, Sigma-Aldrich) assay was used to assess cell sensitivity to paclitaxel, an broad spectrum anticancer drug[7]. The cells were plated in 96-well plates at a concentration of 10^4 cells / 0.1 mL per well. The cells were treated with various concentrations (0-100 μM) of paclitaxel for 24 h. Finally, the culture medium was replaced with MTT solution (5 mg/mL in phenol red or serum-free medium) and incubated for an additional 4 h. Formazan crystals formed by metabolically viable cells were dissolved in 150 μL DMSO. Absorbance at 490 nm was recorded by an Infinite M200 Pro microplate reader (Tecan, Hombrechtikon, Switzerland).

Motility assay

Cell motility was measured by wound healing assay. Monolayer cells in 6-well plates were scratched with a 100 μL pipette tip (t = 0 h) and maintained in 1640 medium containing 1 % FBS. The wounded area was photographed at 0 h and 24 h by a light microscope (Zeiss). Cell mobility was quantified as the difference value between the width of the wound at a given time and the initial width of the wound.

Migration and invasion assays

Falcon cell culture inserts with 8 μm pores (Corning, Cambridge, MA, USA) were placed in 24-well plates. To measure invasion, the upper chamber of a Transwell apparatus was precoated with Matrigel (Corning). Cells in 200 μL culture medium containing 1 % BSA were plated into the upper chamber and 800 μL culture medium containing 20 % FBS was added to the lower chamber. After incubation for 24 h, the noninvasive cells that adhered to the upper chamber were wiped away, while the invasive cells adhered to the lower chamber were fixed with 4 % paraformaldehyde for 30 min and stained with crystal violet for 40 min. The invasive cells per field were assessed by a fluorescence microscope (Zeiss). To measure cell migration, the upper chamber of the inserts was not precoated with Matrigel.

Adhesion assay

To measure cells adhesion to endothelial cells, monolayer HPMECs were precultured in 6-well plates and stimulated with IL-1β (10 ng/mL, Sigma-Aldrich) for 4 h. Subsequently, the target cells labeled with rhodamine 123 (Sigma-Aldrich) came into contact with the surface of HPMECs and oscillated at 200 rpm for 30 min. After that, the cells that didn’t attach to the HPMECs were washed away gently. The cells adhered to HPMECs were photographed and counted by phase-contrast light microscopy (Zeiss). Similarly, to measure adhesion to the ECM, rhodamine 123-labeled target cells were plated into 6-well plates with fibronectin-coated (Sigma-Aldrich) and incubated without shaking for 30 min.

Dual-Luciferase reporter assay

Using the dual-luciferase reporter assay, we determined the transcriptional activity of HIF-1α on the NOL7 promoter sequence with the assistance of easy-to-lab.com (Beijing China). In brief, the DNA sequence of the NOL7 promoter was constructed into the luciferase reporter plasmid (pGL3-basic). The Renilla luciferase plasmid (pRL-TK) and the HIF-1α expressing plasmid (pCDNA3.0-HA-HIF-1α) with pCDNA3.0-HA as control were transfected into 293T

cells through the Lipofectamine 3000 kit. Promoter activity was assessed by the luciferase activity using the Dual-Luciferase Reporter Assay System (Promega). Data were normalized to the results obtained for the internal control Renilla luciferase.

Western blotting

Cell lysates were extracted by RIPA lysis buffer containing 1 μM PMSF at 4℃. Then each equal quality of each sample was separated by SDS-PAGE and transferred to PVDF (Bio-Rad, USA) membranes. After blocking in 5 % skim milk solution for 2 h at room temperature, the PVDF membranes were incubated with dilute primary antibodies overnight at 4°C and secondary antibodies at room temperature for 2 h. Protein blots were visualized using the hypersensitive chemiluminescence kit (Wanlei, Shenyang, China) through ChemiDoc XRS system (Bio-Rad). Protein expression levels were analyzed by Image Lab analysis software 5.0 (Bio-Rad). β-actin was served as the internal control.

Quantitative Real-Time PCR

Total RNA was extracted using TRIzol reagent (Invitrogen), and 100 ng of each sample was then used as template for reverse transcription to cDNA via a PrimeScript® reverse transcription kit (Takara, Japan) in accordance with the manufacturer's guide. PCR was performed by SYBR® Premix Ex Taq™ PCR kit (Takara) on a CFX96™ real-time PCR detection system (Bio-Rad). The threshold cycle values were used to quantify the relative mRNA expression and normalized to β-actin. Primer sequences used are listed in **supplementary Table. 1**.

Mice and *in vivo* mouse study

The mice experiment was guided and approved by the Institutional Animal Care and Use Committee (IACUC) of Fuzhou University, the approval number is SYXK-2019-0007. Female BALB/c nude mice 6-8 weeks old and female C57BL/6 mice 6-8 weeks old were obtained from Slac Animal Inc (Shanghai, China). BALB/c nude mice were housed in clean and sterile cage (Sujingantai Biotechnology, Suzhou,

China) and fed with sterilized rat food (Fuzhou Wushi Animal Center, Fuzhou, China). and sterilized water. C57BL/6 mice were housed in clean cage and fed with usual rat food (Fuzhou Wushi Animal Center) and clean water. All mice were accessed to food and water ad libitum. The animal house was kept in a 12 h-day/night cycle with lights on at 7:00 a.m and in a temperature (26 ± 1°C) and humidity (50 ± 10 %). All mice were randomised into each group by picking random numbers. The mice in all experiments involved in this manuscript were not given any drugs or anesthetic. We have tired our best to minimize animals' pain and suffering.

To obtain the pulmonary metastases of melanoma described above, B16F10 cells (5×10^4 cells in 0.1 mL PBS per mouse, 5 mice per group) were injected into the tail vein of C57BL/6 mice and A375 cells (3×10^6 cells in 0.1 mL PBS per mouse, 5 mice per group) were injected into the tail vein of BALB/c nude mice, respectively. After 7 weeks, all mice were killed by cervical dislocation in a biological safety cabinet (Sujingantai Biotechnology) and the pulmonary metastases were collected for further analysis.

To assess cell metastatic potential, A375-sgNOL7 cells (3×10^6 cells in 0.1 mL PBS per mouse, 5 mice per group) were injected into tail veins of BALB/C nude mice. Mice injected with A375-control cells (3×10^6 cells in 0.1 mL PBS per mouse, 5 mice per group) were used as control group. After 7 weeks, all mice were sacrificed by cervical dislocation and dissected in a biological safety cabinet. The pulmonary metastatic nodules were counted for further analysis. In addition, we assessed the survival time of these two groups of mice; the Kaplan-Meier survival analysis was carried out with the GraphPad Prism software 7.0.

To assess the cell tumorigenicity, A375-sgNOL7 cells (6×10^6 cells in 0.1 mL PBS per mouse, 5 mice per group) were subcutaneously injected into the left axillae of BALB/C nude mice. Mice injected with A375-control cells (6×10^6 cells in 0.1 mL per mouse, 5 mice per group) were used as control group. About 7-10 days later, the subcutaneous tumor grew and tumor volumes were measured every alternate day. After 4 weeks, all mice were killed by cervical dislocation and dissected in a biological safety cabinet. The tumors were collected for further analysis.

Pathological analysis

Excised neoplasms and lung tissues were embedded in paraffin and sliced into sections at thickness of 5 μm. Afterward, histological slices were subjected to hematoxylin and eosin (H&E) staining and imaged by a light microscope (Zeiss).

Immunohistochemistry (IHC)

For validation of the clinical significance of NOL7 in melanoma. Various clinical samples from melanoma patients (normal skin, n = 5; nevus, n = 5; primary melanoma, n = 32; metastases, n = 10) were purchased from Shanghai Zhuoli Biotechnology Co., Ltd (Shanghai, China). NOL7 expression in these samples was evaluated by IHC analysis with the assistance of Shanghai Zhuoli Biotechnology Co., Ltd. The immunostaining score of NOL7 in tumor tissues was quantified by the H-score (histochemical score) based on the staining intensity and heterogeneity under a Nano Zoomer S210 (Hamamatsu, Japan). The percentage of immunostaining and the staining intensity were scored as 0, negative; 1, weak; 2, moderate; and 3, strong. H-score was calculated using the following formula:

H score = (percentage of cells of weak intensity × 1) + (percentage of cells of moderate intensity × 2) + (percentage of cells of strong intensity × 3)

For analysis of NOL7 expression in melanoma formed in mice. NOL7 expression was evaluated by IHC analysis with the assistance of Wuhan Servicebio Co., Ltd. In brief, paraffin embedded sections were fixed in formaldehyde, and a heat-mediated antigen retrieval step in citrate buffer was performed. The samples were blocked in BSA solution for 2 h at 37°C and incubated with anti-NOL7 antibody overnight at 4°C. An horserdish peroxidase conjugated goat anti-rabbit antibody was used as the secondary antibody. NOL7 protein was visualized by AEC staining and examined under the confocal fluorescence microscope (Nikon Eclipse C1, Nikon).

Statistical analysis

Date for all experiments was managed using GraphPad Prism software 7.0 and

represented as the mean ± s.d. A paired t test was used for two group comparison, and a one-way ANOVA was used for multiple group comparison. $P < 0.05$ was considered representative of a significant difference.

Data availability

The datasets generated and / or analyzed during the current study are available from the corresponding author upon reasonable request.

Supplementary Figures

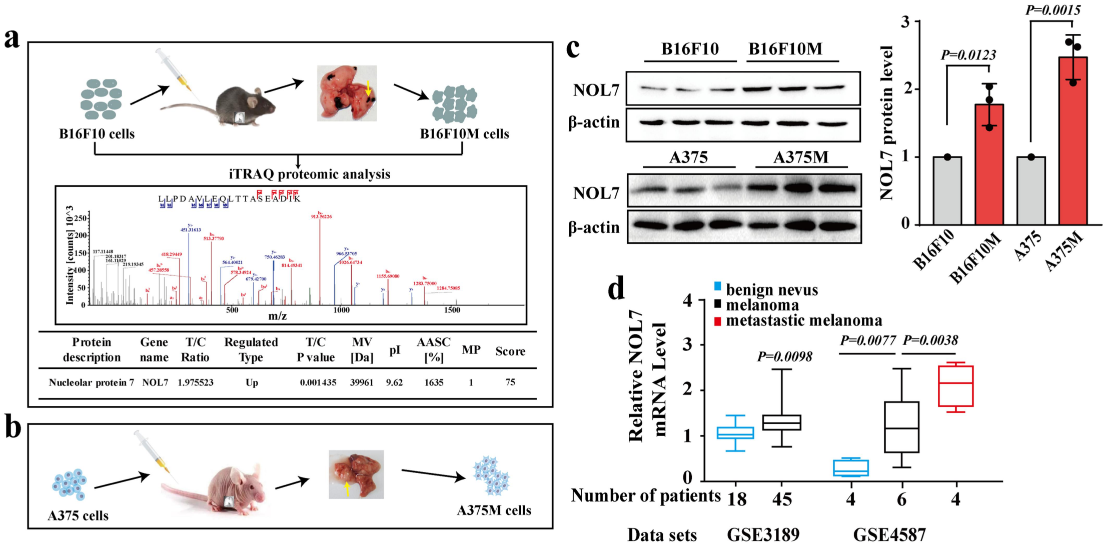

Figure. S1.

NOL7 is up-regulated in metastatic melanoma as compared to primary melanoma.

(a) A flow chart illustrates the establishment of post-metastatic melanoma cell line, B16F10M. Post-metastatic melanoma cell line, B16F10M was derived from pro-metastatic melanoma cells, B16F10 through an animal model of hematogenous tumor metastasis. Global proteomic alterations between B16F10 and B16F10M cells were identified by iTRAQ. Furthermore, mass spectrum peaks for a unique peptide fragment pertaining to NOL7 protein and identification outcomes of NOL7 protein among B16F10 (sample ID: C) and B16F10M (sample ID: T) are shown below. (**b**) A flow chart shows the establishment of post-metastatic melanoma cell line, A375M derived from pro-metastatic melanoma cells, A375 through an animal model of hematogenous tumor metastasis. (**c**) Increased protein expression level of NOL7 in B16F10M and A375M cells relative to B16F10 and A375 cells, respectively, as measured by western blotting. Quantification of NOL7 expression level was shown in right. β-actin was served as the internal control. (**d**) NOL7 expression was increased during the disease progression from normal skin to nevus to primary melanoma as detected by in silico analysis using GSE3189 gene chip (benign nevus n=18, melanoma n=45) and GSE4587 gene chip (Nevus n=4, primary melanoma n=6, metastatic melanoma n=3). Data are presented as mean ± s.d. (n⩾3); t-test.

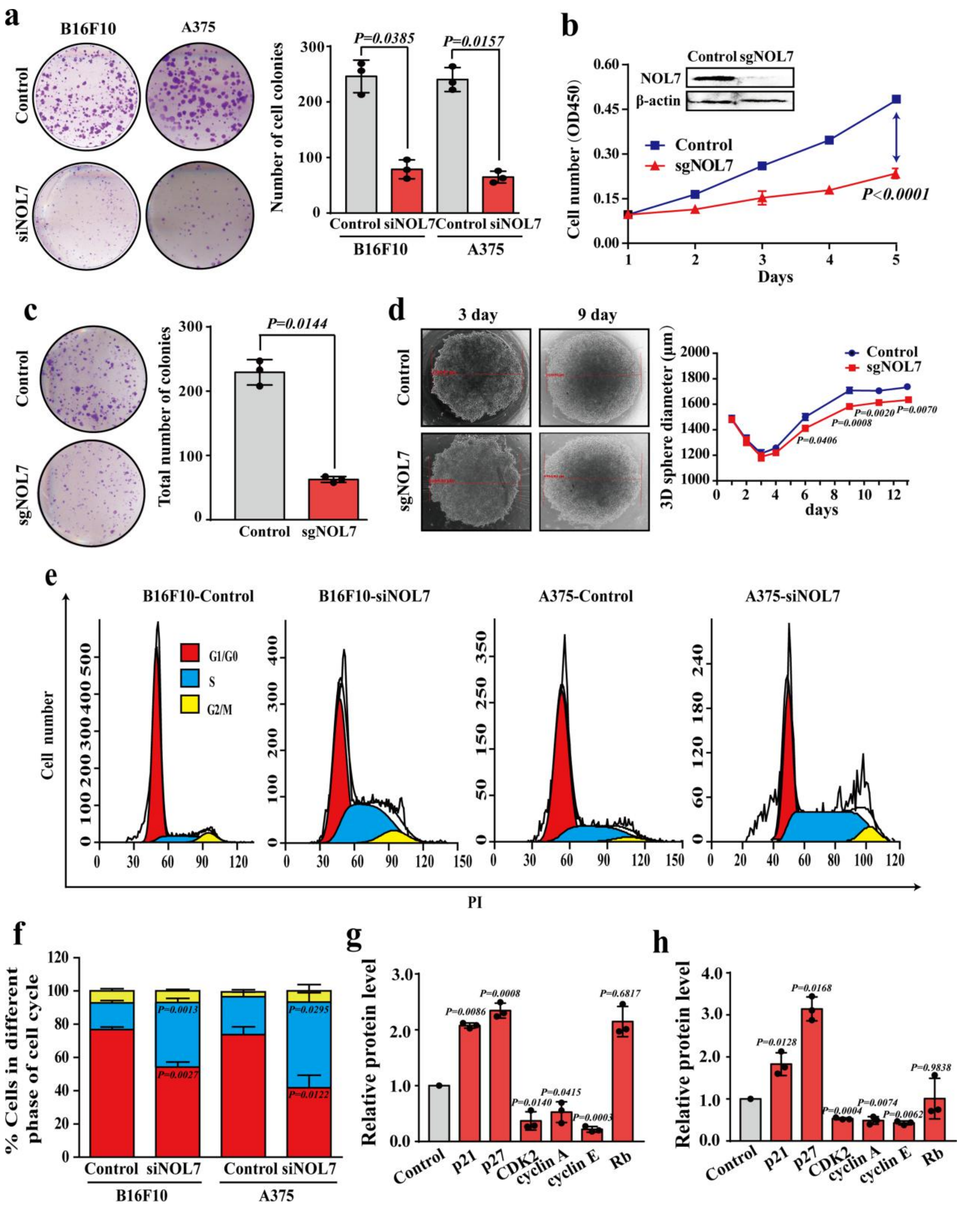

Figure. S2.

NOL7 knockdown/knockout suppresses cell proliferation and induces cell cycle S phase arrest in melanoma cells.

(a) Decreased cell colony formation upon NOL7 knockdown in B16F10 and A375 cells. (**b**) Lower cell proliferation upon NOL7 knockout in A375 cells. Inset shows the considerably depletion of NOL7 expression by CRISPR/Cas9 system in A375 cells. (**c**) Decreased cell colony formation upon NOL7 knockout in A375 cells. (**d**) NOL7 knockout depressed the spherical growth of A375 cells. Cell suspensions formed a

tight sphere on the 3 rd day and continued to grow steadily as spheres. The growth rate of A375-sgNOL7 cell sphere was significantly lower than that of control group. (**e-f**) Flow cytometry scans **(e)** and the quantitative analysis **(f)** shows higher proportion of S phase population upon NOL7 knockdown in B16F10 and A375 cells. (**g-h**) The quantitative analysis for alternation of cell cycle-associated proteins upon NOL7 knockdown in B16F10 cells **(g)** and A375 cells **(h)**, **related to Fig. 1d**. Data are presented as mean ± s.d., for each cohort, n ≥ 3; t-test.

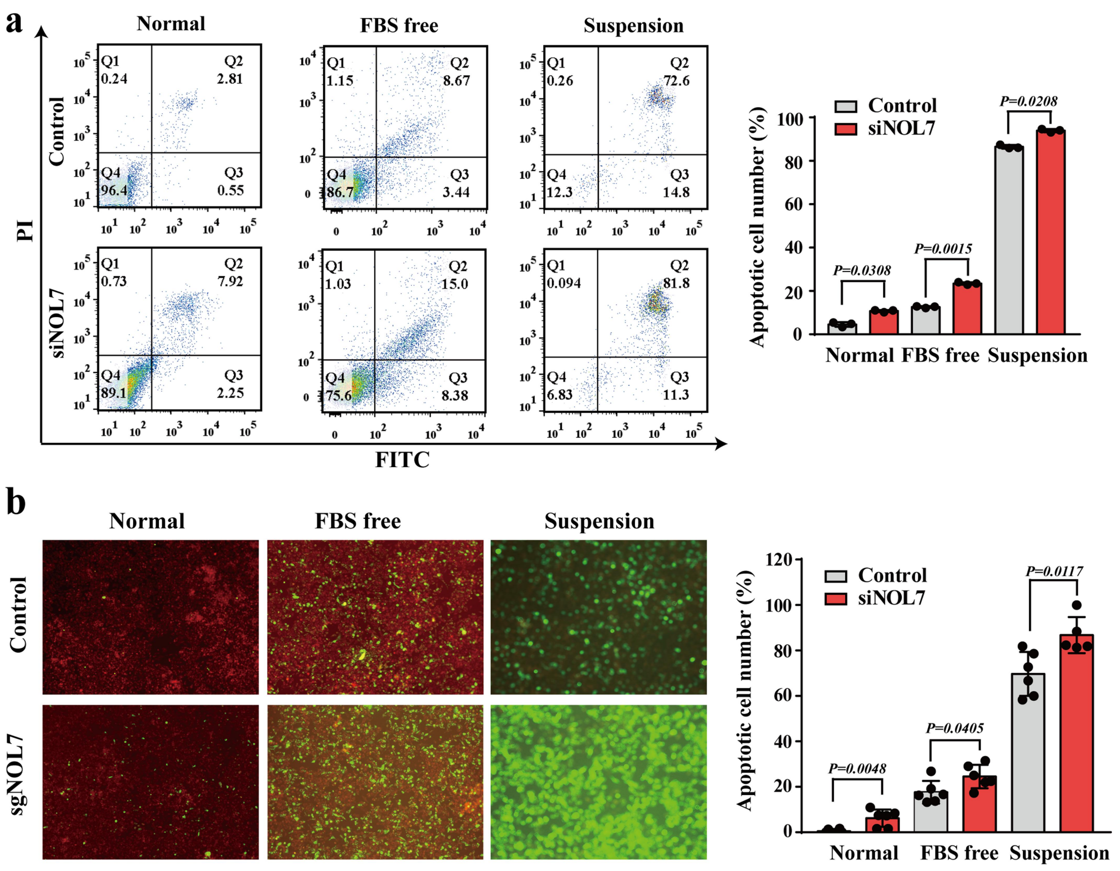

Figure. S3.

NOL7 is associated with lower sensitivity to apoptosis resistance in melanoma cells.

(a) Annexin V-FITC/PI staining demonstrated a higher apoptosis rate upon NOL7 knockdown in A375 cells in FBS-free and nonadherent conditions. Quantification of apoptotic cells was shown in right. (**b**) JC-1 staining demonstrated lower mitochondrial membrane potential upon NOL7 knockout in A375 cells in FBS-free and nonadherent conditions. Quantification of apoptotic cells (the ratio of green fluorescent cell) was shown in right. Data are presented as mean ± s.d. (n ≥ 3); t-test.

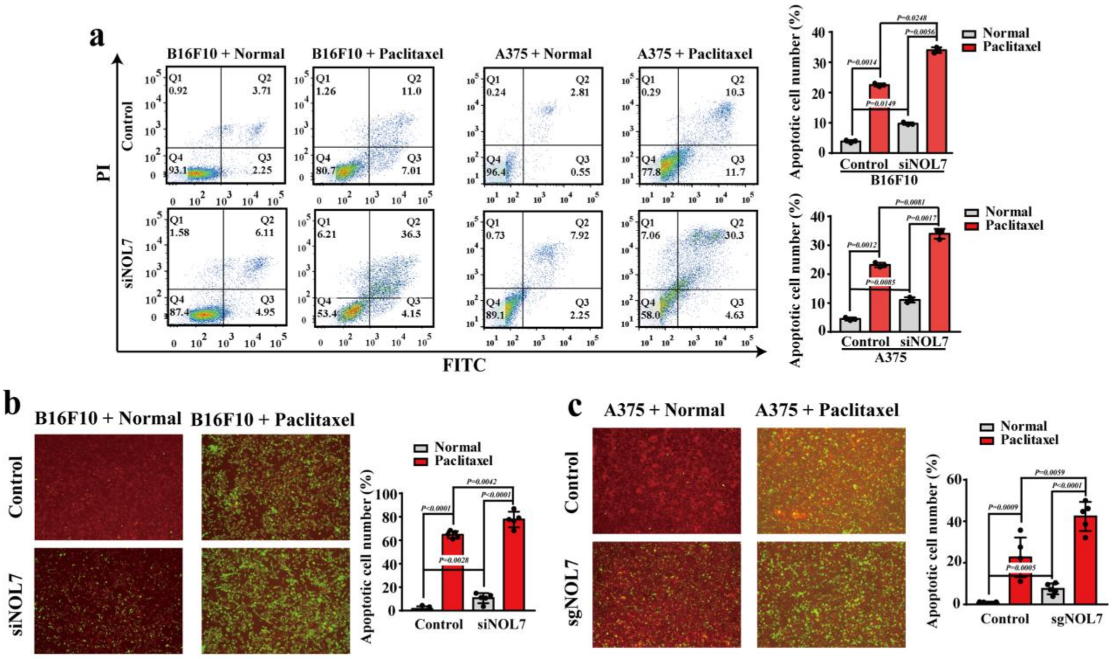

Figure. S4.

NOL7 is associated with lower sensitivity to paclitaxel in melanoma cells.

(a) Annexin V-FITC/PI staining demonstrated a higher apoptosis rate after being treated with 10 μM paclitaxel upon NOL7 knockdown in B16F10 cells and A375 cells. Quantification of apoptotic cells was shown in right. (**b-c**) JC-1 staining demonstrated lower mitochondrial membrane potential after being treated with 10 μM paclitaxel upon NOL7 knockdown in B16F10 cells **(b)** and A375 cells **(c)**. Quantification of apoptotic cells (the ratio of green fluorescent cell) was shown in right. Data are presented as mean ± s.d. (n ≥ 3); t-test.

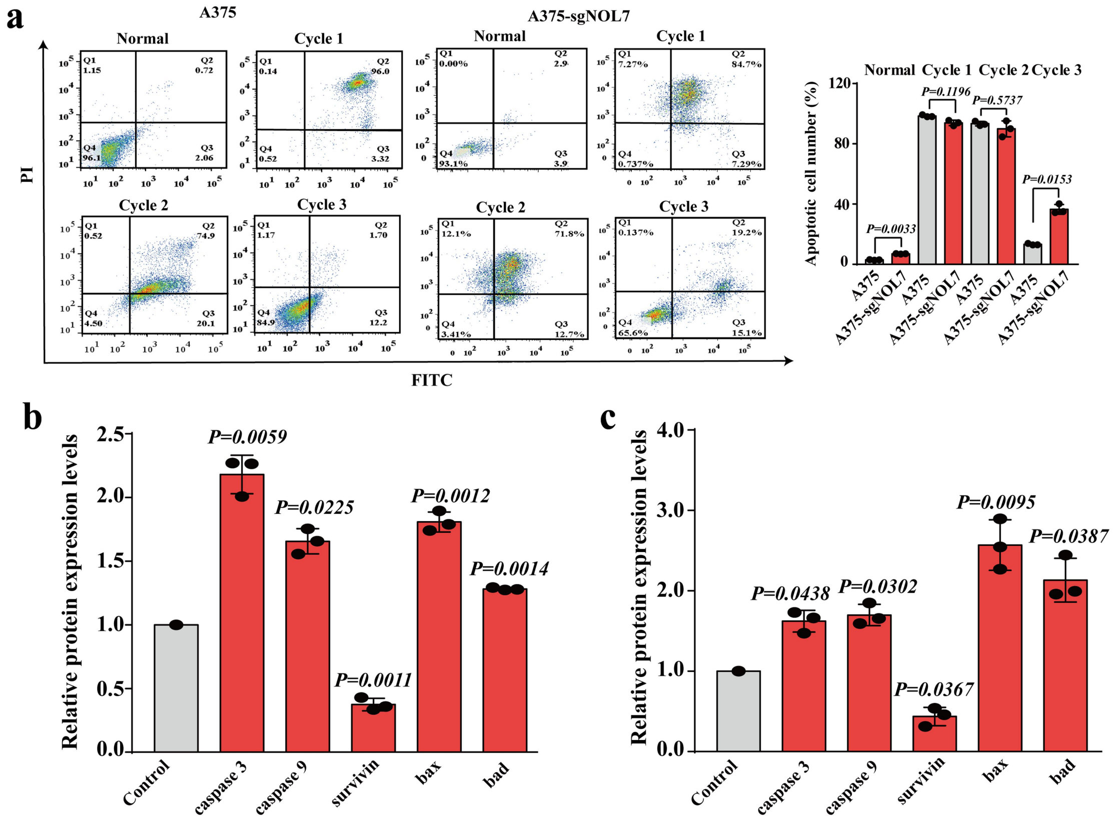

Figure. S5.

NOL7 is associated with lower sensitivity to anoikis in melanoma cells.

(a) Flow cytometry scans showed the generation of anoikis-resistant A375 cells. Quantification of apoptotic cells was shown in right. (**b-c**) The quantitative analysis for alternation of apoptosis-related proteins upon NOL7 knockdown in B16F10 cells **(b)** and A375 cells **(c)**, related to **Fig. 1i**. Data are presented as mean ± s.d. ($n \geq 3$); t-test.

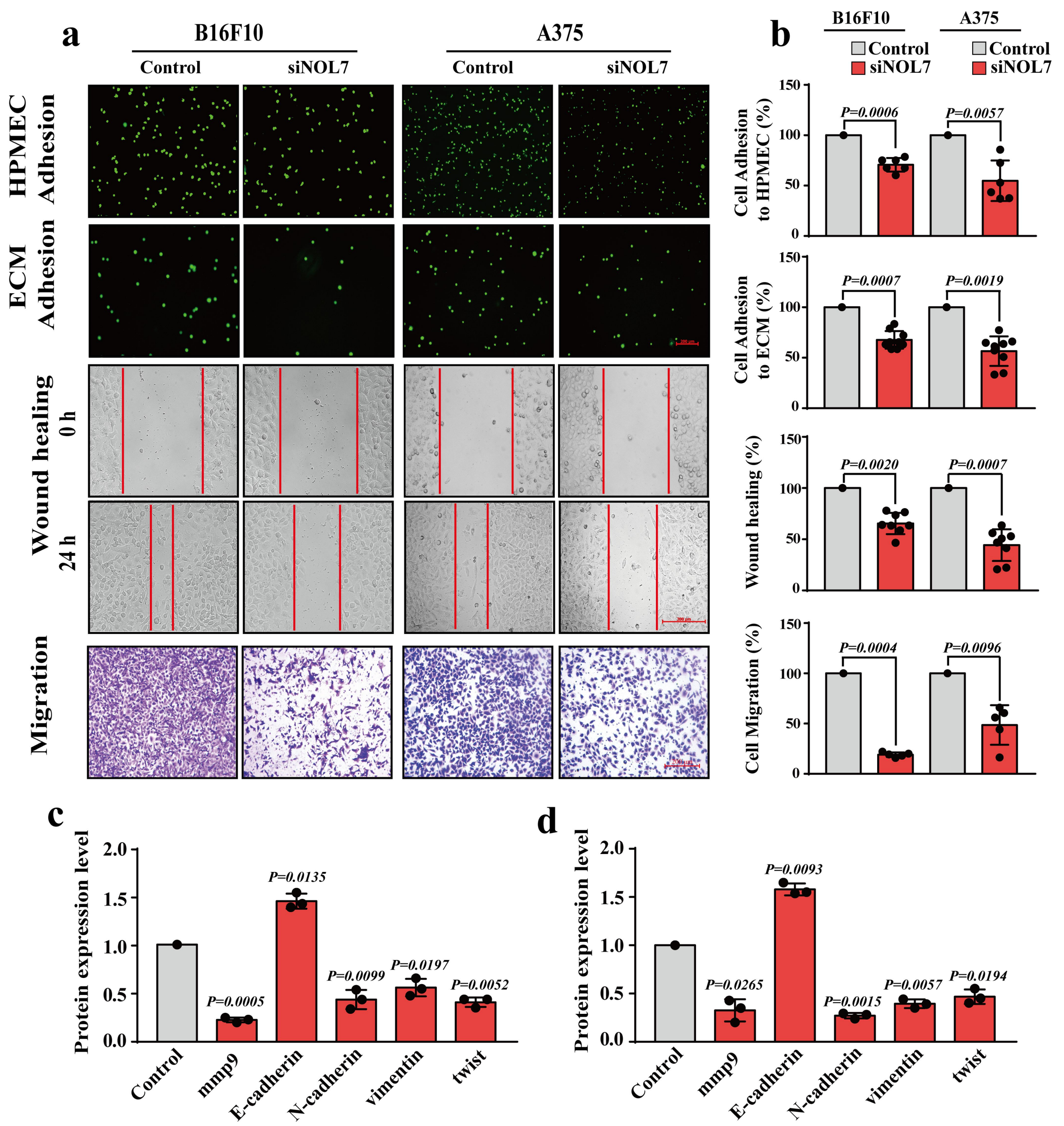

Figure. S6.

NOL7 correlates with metastatic potential of melanoma cells.

(a) Phase micrograph showing adherent melanoma cells (above), melanoma cells during wound healing (middle) and migrating (below) melanoma cells at different time intervals after NOL7 knockdown. (**b**) Quantitative analysis of the effect of NOL7 knockdown on cell adhesion, cell motility and cell migration of B16F10 and A375 cells. (**c-d**) The quantitative analysis for alternation of EMT-related proteins upon NOL7 knockdown in B16F10 cells **(c)** and A375 cells **(d)**, **related to Fig. 1k**. Data are presented as mean ± s.d. (n ≥ 3); t-test.

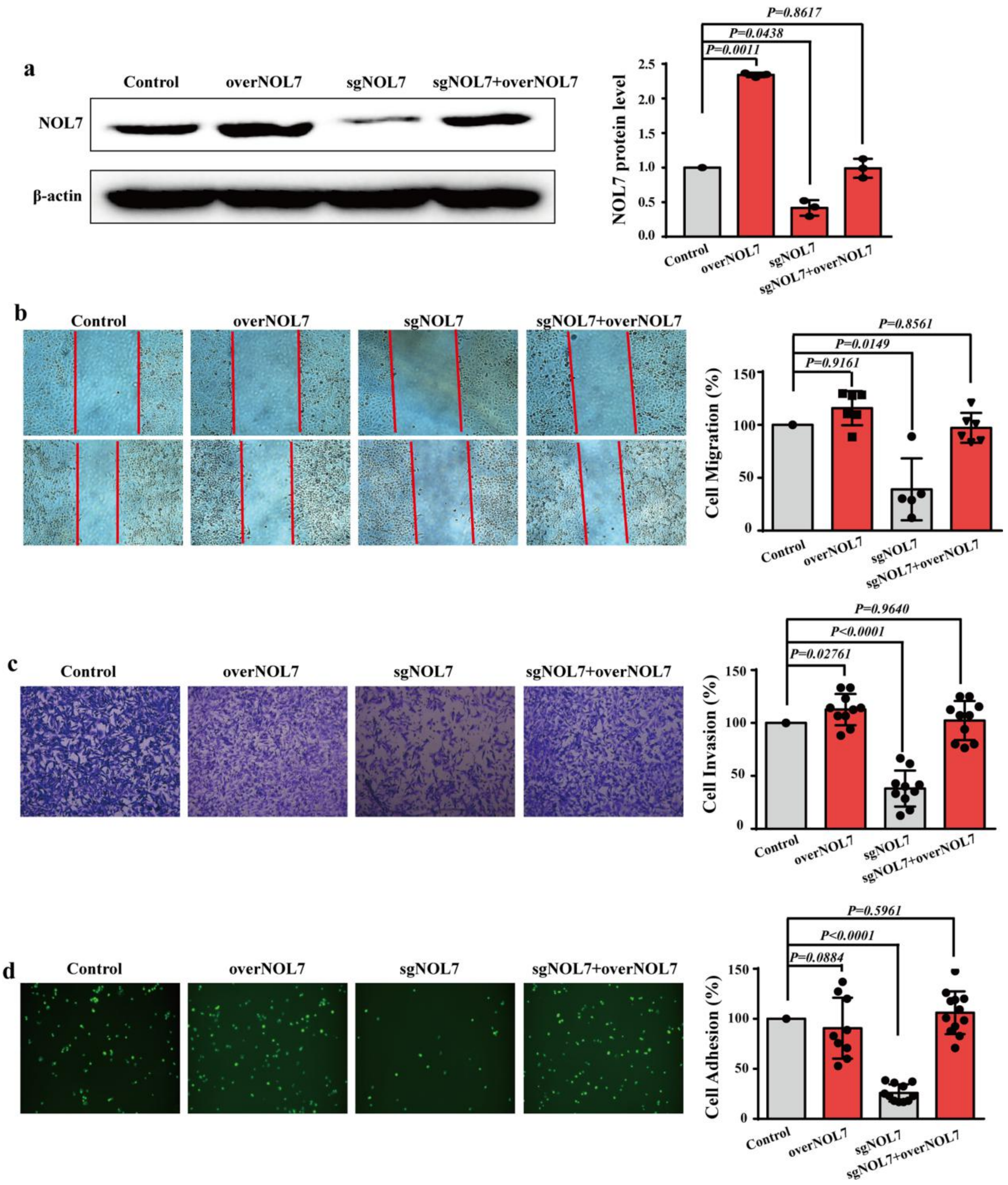

Figure. S7.

NOL7 is necessary for metastatic potential of melanoma cells.

(a) NOL7 expression was significantly altered by being transfected with NOL7 overexpressing plasmids or NOL7 knockout plasmids in A375 cells as detected by western blotting. Quantification of NOL7 protein was shown in right. (**b-d**) Phase micrograph showing melanoma cells during wound healing **(b)**, invading melanoma cells **(c)** and adherent melanoma cells to ECM **(d)** upon NOL7 expression alteration. Quantitative analysis of the effect of NOL7 expression alteration on cell motility, cell migration and cell adhesion to ECM of A375 cells was shown in right. Data are presented as mean ± s.d. (n≥3); t-test.

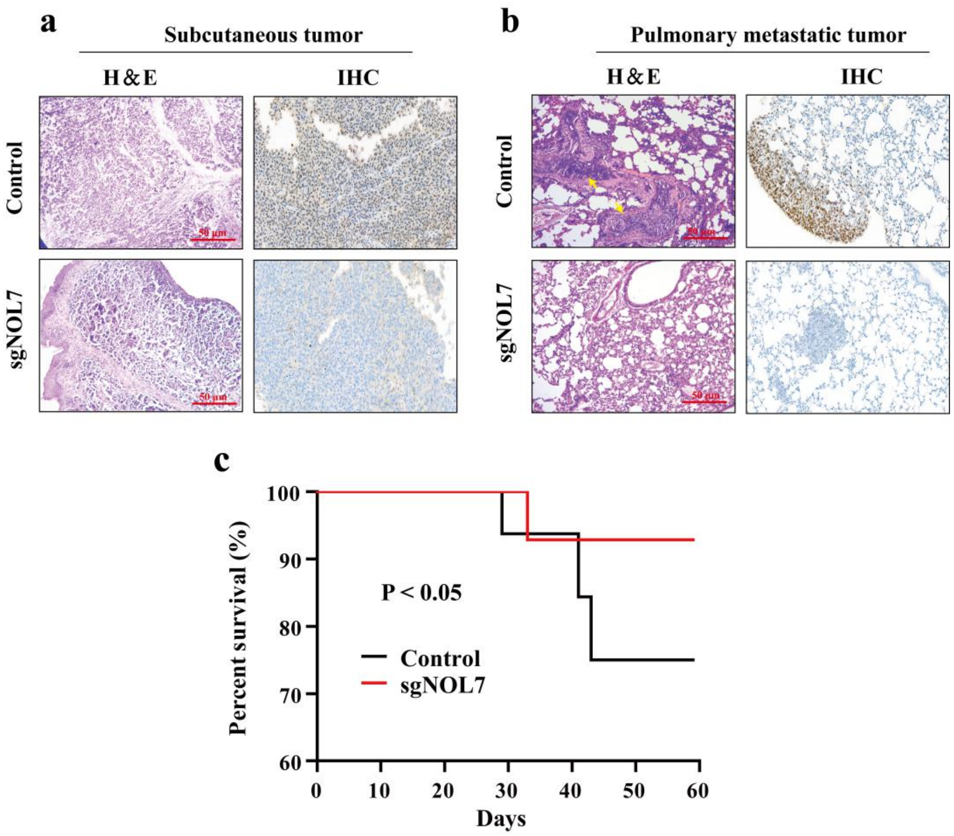

364 **Figure. S8.**

365 **NOL7 is necessary for melanoma growth and metastasis in vivo.**

366 **(a-b)** Representative images of H&E staining and IHC staining of tumors are shown. IHC analysis indicates that NOL7 barely expressed in tumors which were formed by NOL7 knockout cells. (**c**) Overall survival rate of mice injected with control A375 cells and NOL7-knockout A375 cells in caudal veins. Low NOL7 expression is prognostic favourable in mice injected with A375 cells.

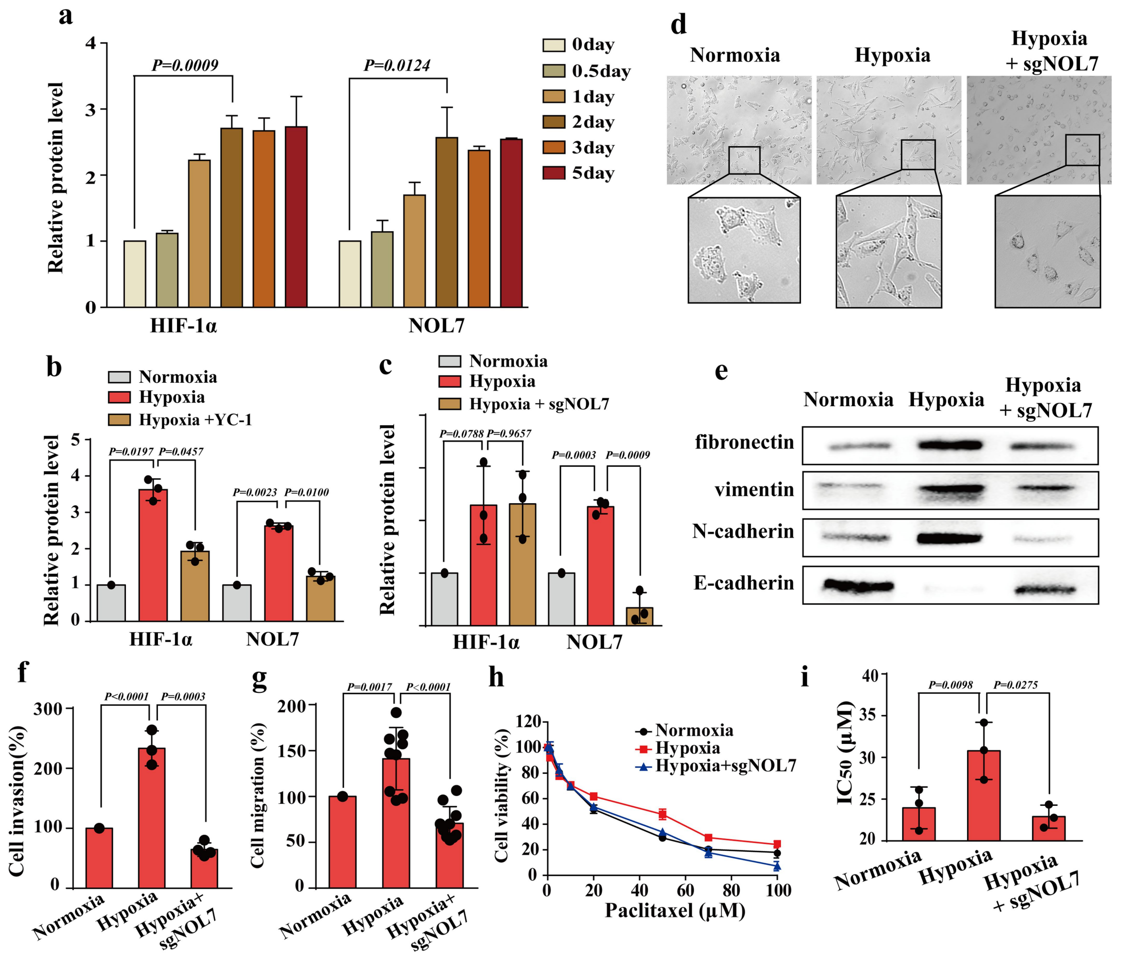

Figure. S9.

NOL7 participates in HIF-1α-induced EMT, cell motility and chemotherapeutic resistance under hypoxia.

(a) Quantitative analysis of HIF-1α and NOL7 expression under hypoxia. **related to Fig. 1l**. (**b**) Quantitative analysis of HIF-1α and NOL7 expression under hypoxia or treatment with YC-1. **related to Fig. 1m**. (**c**) Quantitative analysis of HIF-1α and NOL7 expression under hypoxia or treatment with NOL7 depletion. **related to Fig. 1n**. (**d-e**) NOL7 depletion impaired the cell polarity **(b)** and the expression of EMT markers **(c)** induced by hypoxic condition. (**f-i**) NOL7 depletion repressed hypoxia-induced cell invasion **(f)**, migration **(g)** and drug resistance to paclitaxel **(h-i)**. Data are presented as mean ± s.d. (n ≥ 3); t-test.

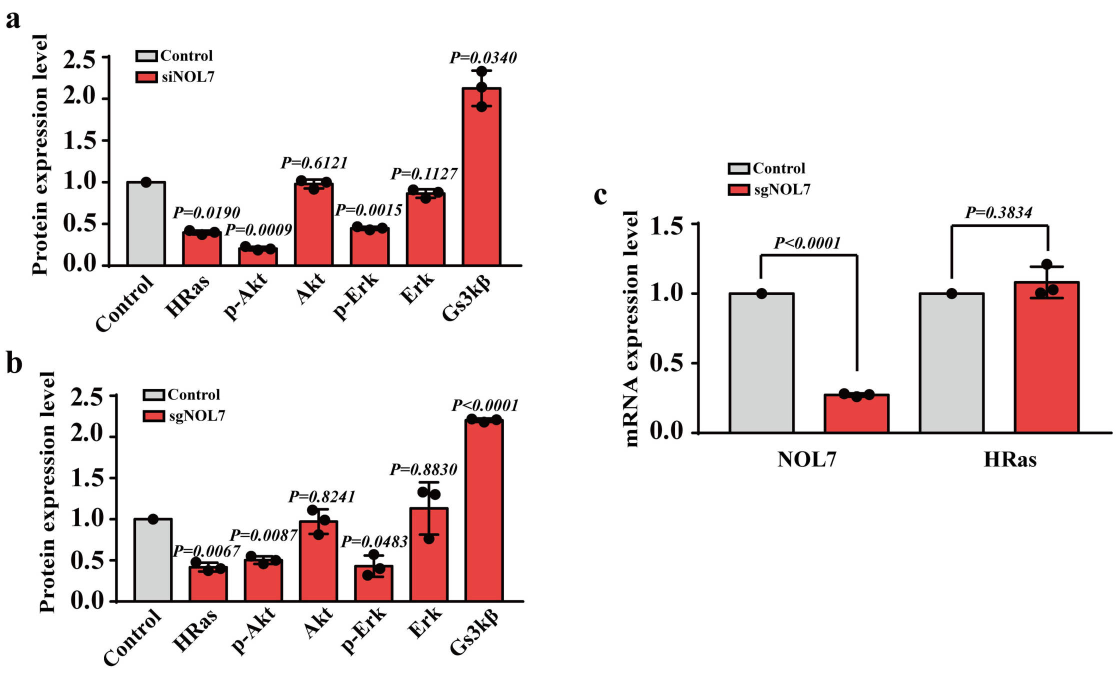

Figure. S10.

NOL7 modulates the PI3K/AKT/ERK pathway in melanoma.

(a-b) The quantitative analysis for alternation of proteins in PI3K/AKT/ERK pathway upon NOL7 knockdown in B16F10 cells **(a)** and A375 cells **(b)**, **related to Fig. 1p**. (**c**) HRas mRNA level was not altered upon NOL7 knockout in A375 cells as detected by qRT-PCR. Data are presented as mean ± s.d. ($n \geq 3$); t-test.

Supplementary Table

Table S1.

Primer sequences used were listed.

Gene	Species	Forward primer sequence (5' → 3')	Reverse primer sequence (5' → 3')
β-actin	human	AGAAAATCTGGCACCACACC	AGAGGCGTACAGGGATAGCA
β-actin	mouse	GGCTGTATTCCCCTCCATCG	CCAGTTGGTAACAATGCCATGT
NOL7	human	TTGCCAACAAGAGGTTACCAG	TCTGACCATCCACCGTCTTTT
NOL7	mouse	GAGTGCTCGCAGGGATAAAAC	GCTGCTCTAATACAGCATCTGG
PCNA	human	GAACTGGTTCATTCATCTCTATGG	TGTCACAGACAAGTAATGTCGATAAA
PCNA	mouse	TTTGAGGCACGCCTGATCC	TTTGAGGCACGCCTGATCC

References:

1 Gerstberger, S. Hafner, M. & Tuschl, T. A census of human RNA-binding proteins. *Nat Rev Genet.* **15,** 829-845 (2014).

2 Hentze, M. W., Castello, A., Schwarzl, T. & Preiss, T. A brave new world of RNA-binding proteins. *Nat Rev Mol Cell Bio.* **19,** 327-341 (2018).

3 Lang, B., Armaos, A. & Tartaglia, G. G. RNAct: Protein-RNA interaction predictions for model organisms with supporting experimental data. *Nucleic Acids Res.* **47,** D601-D606 (2019).

4 Khanna, C. Modeling metastasis in vivo. *Carcinogenesis.* **26,** 513-523 (2004).

5 Li, Y. *et al.* WDR74 modulates melanoma tumorigenesis and metastasis through the RPL5 ‑ MDM2 ‑ p53 pathway. *ONCOGENE* **39** 2741-2755 (2020).

6 Ran, F. A. *et al.* Genome engineering using the CRISPR-Cas9 system. *Nat Protoc.* **8,** 2281-2308 (2013).

7 Guo, Y. Q. *et al.* Efficacy and safety of nab-paclitaxel combined with carboplatin in Chinese patients with melanoma. *Med Oncol.* **32,** 234-240 (2015).
